# Trajectory models of serum creatinine and 28-day mortality in critically ill patients with sepsis complicated by type 2 diabetes mellitus: a cohort study

**DOI:** 10.3389/fendo.2026.1822280

**Published:** 2026-06-23

**Authors:** Shu Yang, Lianzheng Ma, Jinfang Zeng, Yiwen Guo, Chunyi Wu, Xiao Zhang, Minmin Zhu

**Affiliations:** 1 Wuxi School of Medicine, Jiangnan University, Wuxi, China; 2 Department of Anesthesiology and Pain Medicine, Wuxi No. 2 People’s Hospital, Jiangnan University Medical Center, Wuxi, China; 3 School of Medicine, Shanghai Jiao Tong University, Shanghai, China

**Keywords:** 28-day mortality, intensive care unit, MIMIC-IV, sepsis complicated by type 2 diabetes mellitus, serum creatinine

## Abstract

**Background:**

In critically ill patients with sepsis complicated by type 2 diabetes mellitus (T2DM), renal dysfunction is prevalent; however, the association between dynamic serum creatinine trajectory patterns and short-term mortality risk remains inadequately characterized.

**Methods:**

This study was conducted using data from the Medical Information Mart for Intensive Care IV (MIMIC-IV) database. The target population comprised adult intensive care unit (ICU) patients with sepsis complicated by T2DM. Group-Based Trajectory Modeling (GBTM) was employed to categorize the patterns of serum creatinine changes within 72 hours following ICU admission. Multivariable Cox proportional hazards models and Kaplan-Meier (K-M) survival curves were constructed to assess the association between trajectory groups and 28-day all-cause mortality, while restricted cubic spline (RCS) curves were utilized to examine the trend of short-term mortality risk in relation to serum creatinine levels. Furthermore, subgroup analyses and mediation effect analysis of continuous renal replacement therapy (CRRT) were performed.

**Results:**

A total of 1909 patients were included in the study, with 439 deaths occurring within 28 days, corresponding to a 28-day all-cause mortality rate of approximately 23.0%. Three distinct serum creatinine trajectory patterns were identified: low-stable, moderate-increasing, and persistently high. Compared with the low-stable trajectory group, patients in the moderate-increasing and persistently high groups exhibited significantly elevated 28-day mortality risk, which remained statistically significant following multivariable adjustment, with adjustments for age, gender, temperature, platelet count, albumin, blood urea nitrogen, pH, renal disease, severe liver disease, mechanical ventilation, 24-hour urine output, and the Charlson Comorbidity Index (model 2: HR = 1.50, 95% CI: 1.16-1.93, p = 0.02; model 3: HR = 1.46, 95% CI: 1.04-2.05, p = 0.03). RCS analysis revealed a significant nonlinear association between serum creatinine levels and mortality risk. The subgroup analysis results revealed significant interaction effects across gender and CRRT subgroups. Mediation analysis demonstrated that CRRT may exert a partial mediating effect on the association between creatinine elevation and mortality risk.

**Conclusion:**

The early trajectory of serum creatinine changes following ICU admission is independently associated with 28-day mortality in critically ill patients with sepsis complicated by T2DM. Sustained higher serum creatinine levels are significantly associated with an increased risk of mortality.

## 1 Introduction

Sepsis is among the leading causes of mortality in intensive care unit (ICU) patients and is associated with high incidence and case-fatality rates (1). Patients with type 2 diabetes mellitus (T2DM) are particularly vulnerable to sepsis-associated acute kidney injury (S-AKI) (2). This vulnerability may be related to preexisting metabolic dysregulation, impaired immune responses, and reduced renal reserve. Consequently, this population has a higher risk of in-hospital mortality and long-term adverse renal outcomes (3).

Serum creatinine remains a widely used biomarker for assessing kidney function. In S-AKI, serum creatinine often changes dynamically during the early course of critical illness (4). Compared with a single creatinine value, dynamic changes in creatinine may better capture the progression of renal dysfunction. These changes include the magnitude, rate, and duration of creatinine elevation, which have been associated with short-term mortality (5). Although the Kidney Disease: Improving Global Outcomes (KDIGO) criteria are widely used for AKI staging, they rely mainly on threshold-based changes and may not fully capture heterogeneous renal function trajectories in critically ill patients. Therefore, a trajectory-based approach may provide additional prognostic information beyond conventional static measures.

Current clinical evaluation of S-AKI relies largely on peak serum creatinine values or KDIGO staging at a single time point (6). This approach may overlook important information contained in early serial creatinine changes during ICU admission. Patients with similar peak creatinine levels may have different temporal patterns, such as stable, improving, or persistently high trajectories, which may correspond to different risks of adverse outcomes (7). Previous studies have described temporal AKI patterns in sepsis, such as transient, persistent, or recurrent AKI (4). However, most of these studies focused on the general sepsis population.

Importantly, evidence remains limited regarding whether early serum creatinine trajectories can identify distinct mortality-risk subgroups specifically among critically ill patients with both sepsis and T2DM. This subgroup may have unique metabolic and renal vulnerability, but it has not been adequately examined using rigorous trajectory modeling. In addition, continuous renal replacement therapy (CRRT) is commonly used in severe S-AKI. CRRT may reflect both therapeutic intervention and disease severity. However, its role in the association between creatinine trajectories and mortality remains insufficiently understood, particularly in observational critical care data (8).

Leveraging the MIMIC-IV database, this study extracted serial serum creatinine measurements from patients with sepsis and T2DM during the first 72 hours after ICU admission. Group-based trajectory modeling (GBTM) was used to identify distinct creatinine trajectory patterns (1, 9, 10). We then examined whether these trajectory groups were associated with 28-day all-cause mortality. Multivariable Cox proportional hazards models were constructed to evaluate the association between creatinine trajectories and mortality. Restricted cubic splines were used to assess the non-linear association between early creatinine levels and mortality risk. Subgroup analyses were performed to explore whether the associations were consistent across clinically relevant subgroups. Finally, an exploratory mediation analysis was conducted to assess whether CRRT may partly account for the association between creatinine trajectories and mortality, while recognizing that CRRT may also represent disease severity and treatment selection.

This study aims to identify distinct subtypes of early ICU serum creatinine trajectories among patients with sepsis and T2DM using GBTM; assess the independent association between each identified trajectory subtype and 28-day all-cause mortality; and examine whether continuous CRRT moderates the association between creatinine trajectory and mortality—specifically, through a formal mediation analysis. Collectively, these objectives seek to advance dynamic renal function monitoring and refine risk stratification for mortality in this high-risk patient population.

## 2 Materials and methods

### 2.1 Data source

This study was carried out using data extracted from the MIMIC-IV 3.1 database. The first author, Yang Shu, was granted full access to the database and tasked with extracting data related to diabetic patients with sepsis (Credential ID: 62274870). Additionally, this study strictly adheres to the STROBE guidelines.

### 2.2 Participants

**Figure 1:**
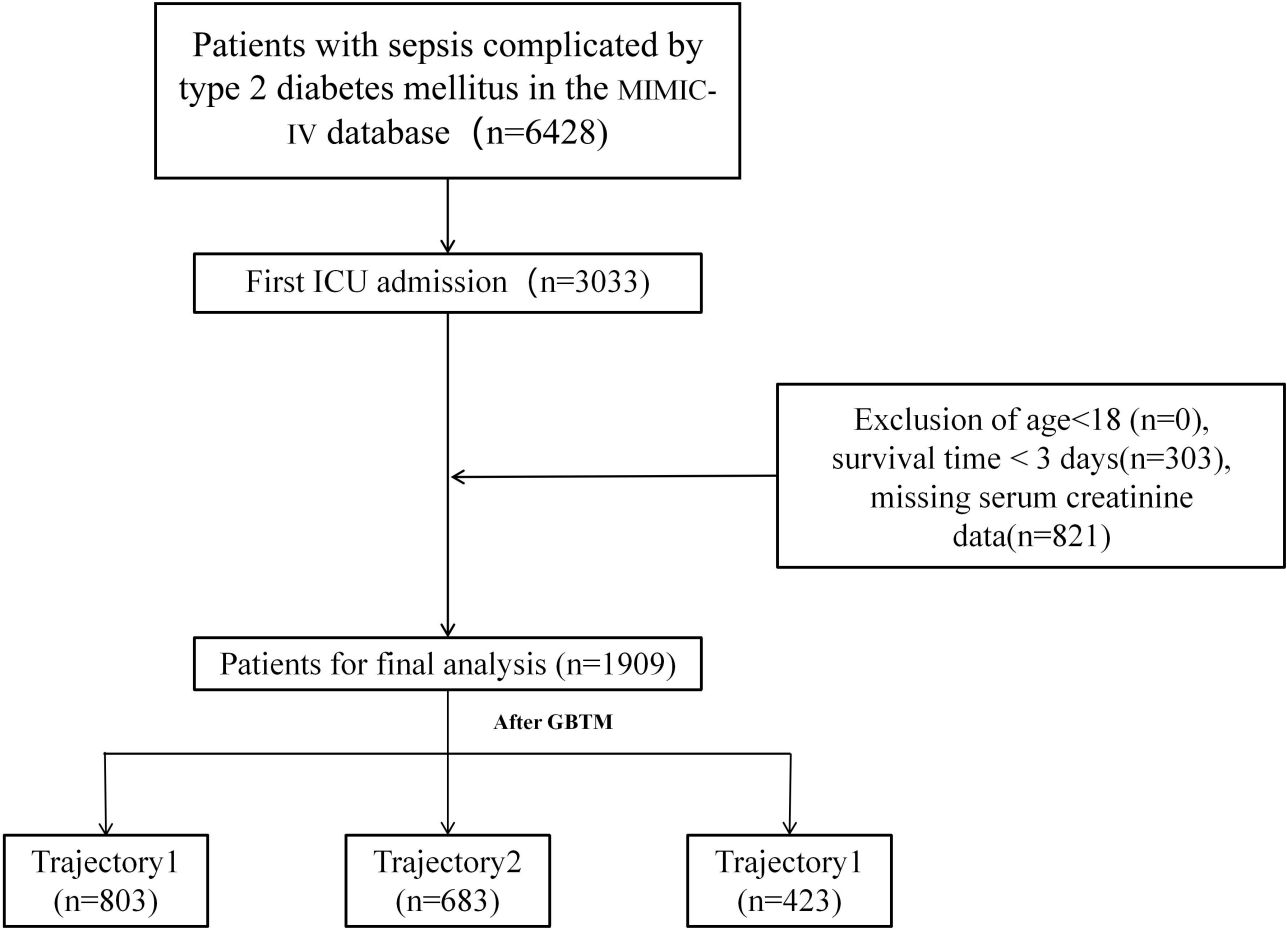
Flow chart of study population inclusion and exclusion.

In this study, cases of sepsis and T2DM were identified using the International Classification of Diseases, 9/10th Revision, Clinical Modification (ICD-9/10-CM) coding systems. The detailed list of codes is provided in **Supplementary Table 1**. Given that this study was conducted using a retrospective database, sepsis was defined based on documented diagnostic codes rather than a prospective bedside Sepsis-3 assessment. While ICD-based definitions are widely employed in MIMIC database studies, this approach may not fully encompass all clinical components specified in the Sepsis-3 definition, such as suspected infection and acute organ dysfunction. The definition of sepsis complicated with T2DM was defined as the concurrent fulfillment of diagnostic criteria for both sepsis and T2DM during the same hospitalization period. Eligibility for study inclusion required simultaneous satisfaction of the following three criteria: (i) First admission to the ICU; (ii) Age ≥ 18 years; (iii) At least one serum creatinine measurement obtained daily within the 3 days prior to ICU admission. Following rigorous screening, a total of 1, 909 patients meeting all inclusion criteria were enrolled in the final analytical cohort (see **Figure 1**). The requirement of at least one daily serum creatinine measurement prior to ICU admission was designed to ensure the adequacy of trajectory estimation. However, this criterion may preferentially enroll patients who received more intensive clinical monitoring or presented with greater disease severity.

### 2.3 Data extraction

This study employed Navicat Premium in conjunction with Structured Query Language (SQL) for data extraction. The extracted data encompassed demographic characteristics, vital signs, laboratory indicators, disease severity scores, comorbidities and clinical interventions. The primary endpoint was 28-day all-cause mortality following ICU admission. Furthermore, this study incorporated CRRT as a potential mediating variable to further investigate its mechanistic role in the prognostic pathway of patients with sepsis complicated by diabetes mellitus.

### 2.4 Covariate selection

Covariates were selected using a combination of the Boruta algorithm and clinical relevance (**Supplementary Figure 1**). Variable selection was conducted by integrating the Boruta algorithm, insights from prior literature, and expert clinical judgment. Variables deemed clinically relevant to sepsis severity, renal dysfunction, and mortality were retained in the multivariable models even if their importance ranking in the Boruta analysis was relatively low. This hybrid strategy was adopted to mitigate the risk of excluding clinically meaningful confounders while minimizing overfitting. Variables confirmed as important by Boruta and considered clinically meaningful were included in the final multivariable model, including age, sex, temperature, platelet count, albumin, BUN, pH, chronic kidney disease, severe liver disease, mechanical ventilation, 24-hour urine output, and Charlson Comorbidity Index.

### 2.5 GBTM

**Figure 2:**
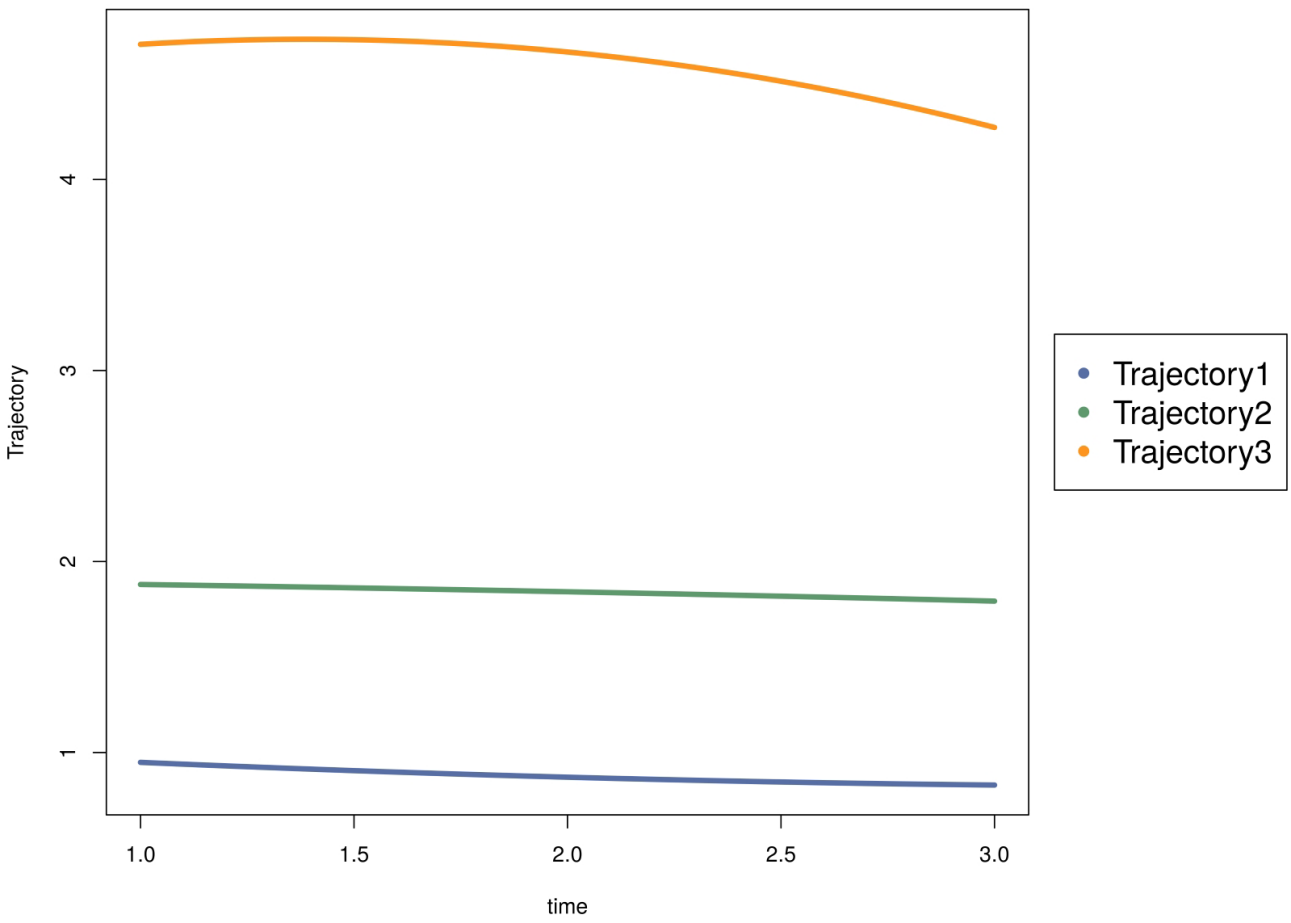
Distinct early serum creatinine trajectory patterns identified by group-based trajectory modeling.

GBTM was employed to identify distinct longitudinal patterns of serum creatinine levels during the early ICU stay. Latent class mixed models were fitted using the lcmm package in R. Creatinine trajectories were modeled via polynomial functions incorporating linear and quadratic time terms, and models with varying numbers of trajectory groups were evaluated (11). To assess the adequacy of the group-based trajectory model, several recommended diagnostic metrics were examined (**Supplementary Table 2**): posterior probability of group membership (average posterior probability, AvePP), odds of correct classification (OCC), estimated group probability (Prob), and the proportion of individuals assigned to each group (Group rate). A well-fitted model is generally characterized by an AvePP > 0.70 and an OCC > 5 (11). In the present study, the AvePP values for the three trajectory groups were 0.957, 0.945, and 0.969, respectively, indicating excellent classification accuracy. The corresponding OCC values were all substantially higher than the recommended threshold, confirming the reliability of individual assignment to their most likely trajectory group. Furthermore, the estimated group probabilities were highly consistent with the observed proportions of participants assigned to each trajectory group, which further supports the adequacy and stability of the final three-group trajectory model (**Figure 2**). More importantly, we compared candidate group-based trajectory models specifying two, three, and four distinct trajectories. The corresponding Bayesian Information Criterion (BIC) values were 15, 576.12, 13, 088.87, and 11, 760.22, respectively—indicating progressively better absolute model fit with increasing complexity. However, the 4-group solution introduced a small, clinically marginal subgroup comprising only 10.6% of the cohort and yielded minimal incremental insight beyond the 3-group structure. In contrast, the 3-group model achieved excellent classification performance (average posterior probabilities: 0.945–0.969; odds of correct classification: 30.2–109.8) while ensuring robust subgroup representation and clear clinical differentiation among trajectories. Given its superior balance of statistical adequacy, parsimony, classification reliability, and substantive interpretability, the 3-group model was selected as the final analytic framework (**Supplementary Table 3**).

### 2.6 Statistical analysis

Baseline characteristics were summarized using descriptive statistics: categorical variables were presented as frequencies (n, %), with intergroup comparisons conducted via the chi-square test or Fisher’s exact test; continuous variables that followed a normal distribution were expressed as mean ± standard deviation, and intergroup differences were assessed using the independent samples t-test; for non-normally distributed continuous variables, values were reported as median, with intergroup comparisons performed using the Wilcoxon rank-sum test. A multivariate Cox proportional hazards regression model was employed to assess the association between serum creatinine trajectory groups and 28-day mortality, the proportional hazards assumption was assessed using Schoenfeld residuals; Kaplan-Meier (K-M) survival curves were generated to compare survival disparities across different trajectory groups; RCS curves were utilized to characterize the dose-response relationship between serum creatinine levels and clinical outcomes, while potential interactions were explored via forest plots; mediation analysis was performed to examine the mediating effect of CRRT. To assess the robustness of the identified creatinine trajectory patterns, a sensitivity analysis was conducted using serum creatinine measurements collected within the first 7 days following ICU admission. Group-based trajectory modeling was then repeated with identical modeling strategies and selection criteria to ensure methodological consistency. Variables with over 40% missing data were excluded from the analysis. For the remaining variables, missing values were addressed via multiple imputation by chained equations (MICE). Five imputed datasets were generated and pooled in accordance with Rubin’s rules. All statistical analyses were performed using R software (version 4.1.2) and Free Statistics software (version 1.9). A two-tailed P value < 0.05 was considered statistically significant.

## 3 Results

### 3.1 Baseline characteristics of study individuals

A total of 1, 909 patients were enrolled in this study, with 803 cases (42.1%) assigned to Trajectory 1, 683 cases (35.6%) to Trajectory 2, and 423 cases (22.2%) to Trajectory 3 (**Table 1**). Among these groups, Trajectory 3 exhibited the highest proportion of male patients. Patients in the higher trajectory groups were more likely to present with comorbid kidney disease and oliguria. Notably, the proportion of patients undergoing CRRT and mechanical ventilation was significantly higher in Trajectory 3, which may indicate a more severe clinical status. Specifically, among patients receiving CRRT, the intergroup differences in proportions were statistically significant. Patients in the lower trajectory groups had lower levels of WBC count, anion gap, BUN, potassium, INR, and PT. As the creatinine trajectory transitioned from the stable group (Trajectory 1) to the increasing group (Trajectory 3), patients showed a heavier disease burden and greater treatment requirements, further suggesting that distinct creatinine trajectories may reflect varying degrees of clinical severity.

**Table 1:** Baseline characteristics of patients according to creatinine trajectory groups.

Variables	Total (n = 1909)	Trajectory 1 (n = 803)	Trajectory 2 (n = 683)	Trajectory 3 (n = 423)	p
Demographics					
Age(year)	68.0 (59.0, 77.0)	66.0 (56.5, 77.0)	71.0 (62.0, 79.0)	67.0 (58.0, 74.5)	< 0.001
Gender, n (%)					< 0.001
Female	754 (39.5)	392 (48.8)	242 (35.4)	120 (28.4)	
Male	1155 (60.5)	411 (51.2)	441 (64.6)	303 (71.6)	
Marita status, n (%)					0.059
Married	792 (41.5)	325 (40.5)	294 (43)	173 (40.9)	
Single	536 (28.1)	215 (26.8)	181 (26.5)	140 (33.1)	
Windowed	239 (12.5)	105 (13.1)	94 (13.8)	40 (9.5)	
Divorced	342 (17.9)	158 (19.7)	114 (16.7)	70 (16.5)	
Insurance, n (%)					0.117
Medicare	1240 (65.0)	500 (62.3)	472 (69.1)	268 (63.4)	
Private	344 (18.0)	155 (19.3)	105 (15.4)	84 (19.9)	
Medicaid	271 (14.2)	127 (15.8)	87 (12.7)	57 (13.5)	
Other	54 (2.8)	21 (2.6)	19 (2.8)	14 (3.3)	
Race, n (%)					0.021
White	1143 (59.9)	490 (61)	417 (61.1)	236 (55.8)	
Black	269 (14.1)	97 (12.1)	91 (13.3)	81 (19.1)	
Asian	71 (3.7)	35 (4.4)	28 (4.1)	8 (1.9)	
Hispanic/Latino	73 (3.8)	34 (4.2)	25 (3.7)	14 (3.3)	
Other/Unknown	353 (18.5)	147 (18.3)	122 (17.9)	84 (19.9)	
Vital signs					
24-hour urine output (ml/kg/h)	0.7 (0.3, 2.2)	1.2 (0.6, 3.2)	0.6 (0.3, 1.7)	0.3 (0.2, 0.8)	< 0.001
Heart rate (beats/minute)	107.0 (93.0, 123.0)	110.0 (97.0, 124.0)	106.0 (92.0, 122.0)	103.0 (88.0, 122.0)	< 0.001
Systolic blood pressure (mmHg)	86.0 (77.0, 95.0)	89.0 (80.0, 97.0)	85.0 (76.0, 94.0)	84.0 (73.0, 92.0)	< 0.001
Diastolic blood pressure (mmHg)	44.0 (38.0, 50.0)	45.0 (39.0, 52.0)	43.0 (37.0, 49.0)	42.0 (36.0, 48.0)	< 0.001
Mean arterial pressure (mmHg)	56.0 (50.0, 63.0)	58.0 (52.0, 64.0)	56.0 (49.0, 62.0)	55.0 (49.0, 60.0)	< 0.001
Respiratory rate (breath/minute)	29.0 (25.0, 33.0)	29.0 (25.0, 34.0)	29.0 (25.0, 33.0)	28.0 (24.0, 33.0)	0.07
Temperature (°C)	36.5 (36.3, 36.7)	36.6 (36.3, 36.8)	36.5 (36.2, 36.7)	36.4 (36.0, 36.7)	< 0.001
SpO2 (%)	92.0 (89.0, 94.0)	92.0 (90.0, 95.0)	92.0 (89.0, 94.0)	92.0 (89.0, 94.0)	0.097
Laboratory indicators					
Glucose (g/dL)	172.5 (136.0, 219.6)	173.0 (136.7, 219.2)	174.1 (141.4, 221.1)	163.5 (124.2, 216.9)	0.012
Hematocrit (%)	29.3 (25.1, 33.9)	29.9 (25.6, 34.3)	29.0 (25.2, 34.0)	28.5 (24.3, 32.6)	< 0.001
Hemoglobin (g/dL)	9.5 (8.1, 11.0)	9.8 (8.3, 11.3)	9.3 (8.0, 11.0)	9.1 (7.7, 10.6)	< 0.001
Platelet (10(9)/L)	173.0 (114.0, 243.0)	181.0 (122.0, 251.0)	168.0 (109.0, 236.5)	165.0 (105.5, 237.0)	0.002
WBC (109/L)	15.8 (10.9, 21.9)	14.8 (10.6, 20.5)	16.4 (11.1, 22.8)	16.7 (11.6, 22.4)	< 0.001
Albumin(g/dL)	2.9 (2.6, 3.3)	2.9 (2.6, 3.3)	2.9 (2.6, 3.3)	2.9 (2.5, 3.2)	0.074
Anion gap (mmol/L)	18.0 (15.0, 21.0)	16.0 (14.0, 19.0)	18.0 (16.0, 22.0)	21.0 (18.0, 25.0)	< 0.001
Bicarbonate (mmol/L)	19.0 (16.0, 22.0)	20.0 (18.0, 23.0)	19.0 (15.0, 22.0)	18.0 (14.0, 21.0)	< 0.001
BUN (mg/dL)	37.0 (23.0, 58.0)	22.0 (16.0, 32.0)	43.0 (31.0, 58.0)	67.0 (49.0, 91.5)	< 0.001
Calcium (mg/dL)	7.9 (7.4, 8.4)	7.8 (7.3, 8.3)	7.9 (7.4, 8.4)	7.9 (7.3, 8.5)	0.348
Chloride (mmol/L)	105.0 (101.0, 109.0)	105.0 (102.0, 109.0)	105.0 (101.5, 110.0)	102.0 (97.0, 107.0)	< 0.001
Sodium (mmol/L)	139.0 (136.0, 142.0)	139.0 (136.0, 142.0)	140.0 (137.0, 143.0)	138.0 (135.0, 141.0)	< 0.001
Potassium (mmol/L)	4.6 (4.1, 5.2)	4.3 (4.0, 4.8)	4.7 (4.3, 5.3)	5.0 (4.5, 5.7)	< 0.001
INR	1.5 (1.3, 1.9)	1.4 (1.2, 1.7)	1.5 (1.3, 2.0)	1.5 (1.3, 2.1)	< 0.001
INR (seconds)	15.9 (13.8, 20.5)	15.4 (13.6, 18.8)	16.5 (14.0, 21.6)	16.2 (14.1, 22.8)	< 0.001
PTT (seconds)	34.5 (29.3, 48.4)	32.9 (28.2, 45.0)	34.9 (29.4, 49.5)	37.9 (31.1, 53.5)	< 0.001
ALT (IU/L)	36.0 (20.0, 85.0)	35.0 (20.0, 75.0)	34.0 (20.0, 81.0)	38.0 (20.0, 115.8)	0.062
ALP (IU/L)	111.0 (78.0, 168.0)	109.0 (78.0, 165.0)	105.0 (74.5, 158.4)	119.0 (84.5, 187.6)	< 0.001
AST (IU/L)	57.0 (30.0, 129.3)	53.0 (28.0, 108.1)	58.1 (32.0, 129.4)	73.0 (33.0, 196.0)	< 0.001
Total Bilirubin (mg/dL)	0.9 (0.5, 1.9)	0.9 (0.5, 1.6)	0.9 (0.5, 2.0)	1.0 (0.5, 2.3)	0.137
Lactate (mmol/L)	2.4 (1.7, 3.6)	2.2 (1.7, 3.1)	2.7 (1.8, 4.0)	2.4 (1.7, 3.8)	< 0.001
PH	7.3 (7.3, 7.4)	7.3 (7.3, 7.4)	7.3 (7.2, 7.4)	7.3 (7.2, 7.4)	< 0.001
Severity scores					
OASIS	36.0 (29.0, 44.0)	34.0 (28.0, 41.0)	38.0 (31.0, 45.0)	40.0 (32.0, 48.0)	< 0.001
Charlson Comorbidity Index	8.0 (6.0, 10.0)	6.0 (5.0, 8.0)	8.0 (6.0, 10.0)	9.0 (7.0, 10.0)	< 0.001
APSIII	62.0 (48.0, 83.0)	50.0 (40.0, 66.0)	67.0 (53.0, 88.0)	76.0 (61.0, 100.5)	< 0.001
SAPSII	43.0 (35.0, 53.0)	37.0 (30.0, 46.0)	46.0 (38.0, 55.5)	51.0 (42.0, 61.0)	< 0.001
Comorbidities					
Myocardial infarct, n (%)	431 (22.6)	131 (16.3)	181 (26.5)	119 (28.1)	< 0.001
Congestive heart failure, n (%)	807 (42.3)	246 (30.6)	331 (48.5)	230 (54.4)	< 0.001
Peripheral vascular disease, n (%)	249 (13.0)	81 (10.1)	100 (14.6)	68 (16.1)	0.004
Cerebrovascular disease, n (%)	249 (13.0)	102 (12.7)	80 (11.7)	67 (15.8)	0.131
Chronic pulmonary disease, n (%)	500 (26.2)	219 (27.3)	185 (27.1)	96 (22.7)	0.179
Mild liver disease, n (%)	324 (17.0)	117 (14.6)	110 (16.1)	97 (22.9)	< 0.001
Renal disease, n (%)	742 (38.9)	91 (11.3)	331 (48.5)	320 (75.7)	< 0.001
Malignant cancer, n (%)	281 (14.7)	140 (17.4)	88 (12.9)	53 (12.5)	0.017
Severe liver disease, n (%)	151 (7.9)	50 (6.2)	58 (8.5)	43 (10.2)	0.041
Metastatic solid tumor, n (%)	129 (6.8)	69 (8.6)	39 (5.7)	21 (5)	0.022
Treatment					
Aspirin, n (%)	444 (23.3)	151 (18.8)	190 (27.8)	103 (24.3)	< 0.001
Vasoactive, n (%)	1000 (52.4)	356 (44.3)	389 (57)	255 (60.3)	< 0.001
Mechanical ventilation, n (%)	231 (12.1)	81 (10.1)	85 (12.4)	65 (15.4)	0.025
CRRT, n (%)	279 (14.6)	28 (3.5)	86 (12.6)	165 (39)	< 0.001

SpO2, Saturation of peripheral oxygen; WBC, White Blood Cell; BUN, Blood Urea Nitrogen; INR, International Normalized Ratio; APSIII, Acute physiology score III; SAPSII, Simplified acute physiology score II; OASIS, Oxford acute severity of illness score.

Bold text indicates category headings used for organizational purposes only and does not denote statistical significance.

### 3.2 Association between serum creatinine and 28-day mortality

Cubic spline analysis (**Supplementary Figure 2**) revealed a significant nonlinear association between the average serum creatinine level during the first 3 days of ICU admission and 28-day mortality (P for non−linearity: 0.003). Using a reference value of approximately 1.4 mg/dL, the adjusted mortality risk increased progressively with elevated creatinine levels, particularly within the range of 1.5 to 3.0 mg/dL. This finding indicates that even moderately elevated serum creatinine levels may be associated with a significantly increased risk of short-term mortality. At higher creatinine levels, the risk curve plateaued, accompanied by a wider confidence interval, which may reflect reduced sample size and clinical heterogeneity. In the Cox proportional hazards regression model, distinct creatinine trajectory groups were significantly associated with 28-day mortality (**Table 2**). In the unadjusted model (Model 1), relative to the Trajectory 1 group, patients in the Trajectory 2 and Trajectory 3 groups exhibited a significantly elevated 28-day mortality risk (HR = 1.81, 95% CI: 1.44–2.29, P < 0.001;HR= 2.29, 95% CI: 1.78–2.94, P < 0.001). This indicates that patients with higher or persistently abnormal creatinine trajectories have a significantly increased short-term mortality risk compared to those with low and stable trajectories. Notably, the risk of death increases substantially with higher levels of creatinine trajectory changes, suggesting that dynamic changes in kidney function may help identify high-risk patients. In the further adjusted model (Model 2), the aforementioned associations remained statistically significant (Trajectory 2: HR = 1.53, 95% CI: 1.20–1.95, P = 0.001;Trajectory 3: HR = 1.50, 95% CI: 1.10–2.04, P = 0.011). In the fully adjusted model (Model 3), the association between creatinine trajectory groups and 28-day mortality remained robust (Trajectory 2: HR = 1.50, 95% CI: 1.16–1.93, P = 0.002; Trajectory 3: HR = 1.46, 95% CI: 1.04–2.05, P = 0.030). Even after adjusting for relevant confounding factors, higher creatinine trajectories remained associated with an approximately 50% increase in short-term mortality risk, indicating that dynamic changes in creatinine may hold independent prognostic value. Multivariable-adjusted results revealed that patients with higher creatinine change trajectories (Trajectories 2 and 3) exhibited a significantly elevated short-term mortality risk relative to those with low and stable trajectories (Trajectory 1). These findings further support the proposition that dynamic creatinine trajectories may be more conducive to early risk stratification and identification of high-risk patients compared to single-point creatinine measurements.

**Table 2:** Multivariable Cox proportional hazards models for 28-day mortality according to creatinine trajectory groups.

Variables	Model 1		Model 2		Model 3	
	HR(95% CI)	*P*-value	HR(95% CI)	*P*-value	HR(95% CI)	*P*-value
28-day mortality						
Creatinine trajectories						
Trajectory 1	1 (Reference)		1 (Reference)		1 (Reference)	
Trajectory 2	1.81 (1.44~2.29)	<0.001	1.53 (1.2~1.95)	0.001	1.5 (1.16~1.93)	0.002
Trajectory 3	2.29 (1.78~2.94)	<0.001	1.5 (1.1~2.04)	0.011	1.46 (1.04~2.05)	0.03
*P* for trend		<0.001		0.006		0.022

Model 1: No Adjusted.

Model 2: Adjusted for age、gender、temperature、platelets、albumin、BUN and PH.

Model 3: Adjusted for Model 2 Plus renal disease、severe liver disease、mechanical ventilation、24-hour urine output and charlson comorbidity index.

### 3.3 Cumulative mortality curve analysis

**Figure 3:**
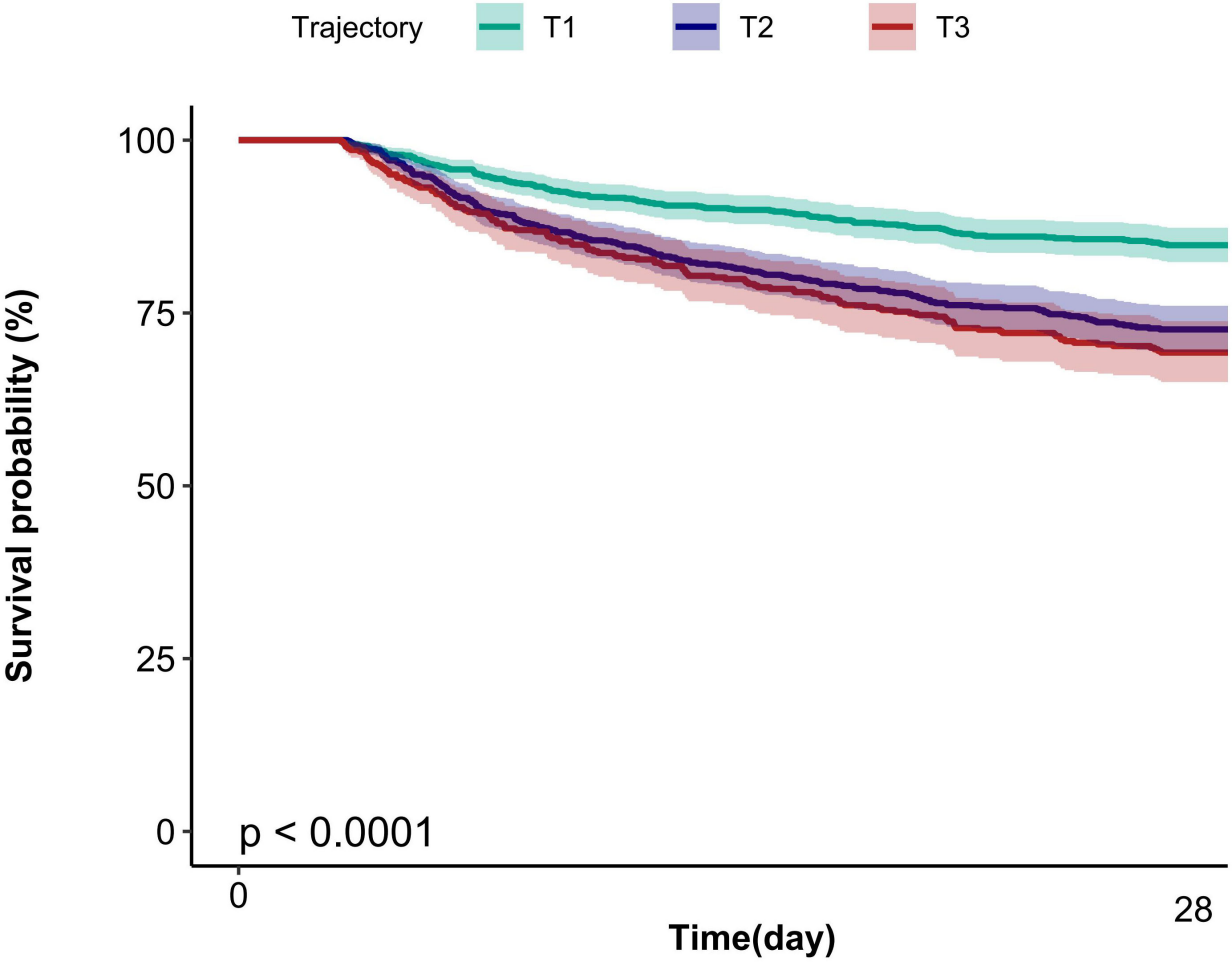
Kaplan–Meier survival curves for 28-day mortality according to creatinine trajectory groups.

Kaplan-Meier curves grouped by trajectory patterns (**Figure 3**) revealed statistically significant differences in 28-day survival rates across the groups (p < 0.0001). Specifically, the low-creatinine trajectory group exhibited the highest survival rate, while the high-creatinine trajectory group had the lowest survival rate. These findings indicate a consistent prognostic disparity between trajectory patterns and survival outcomes.

### 3.4 Subgroup analysis

**Figure 4:**
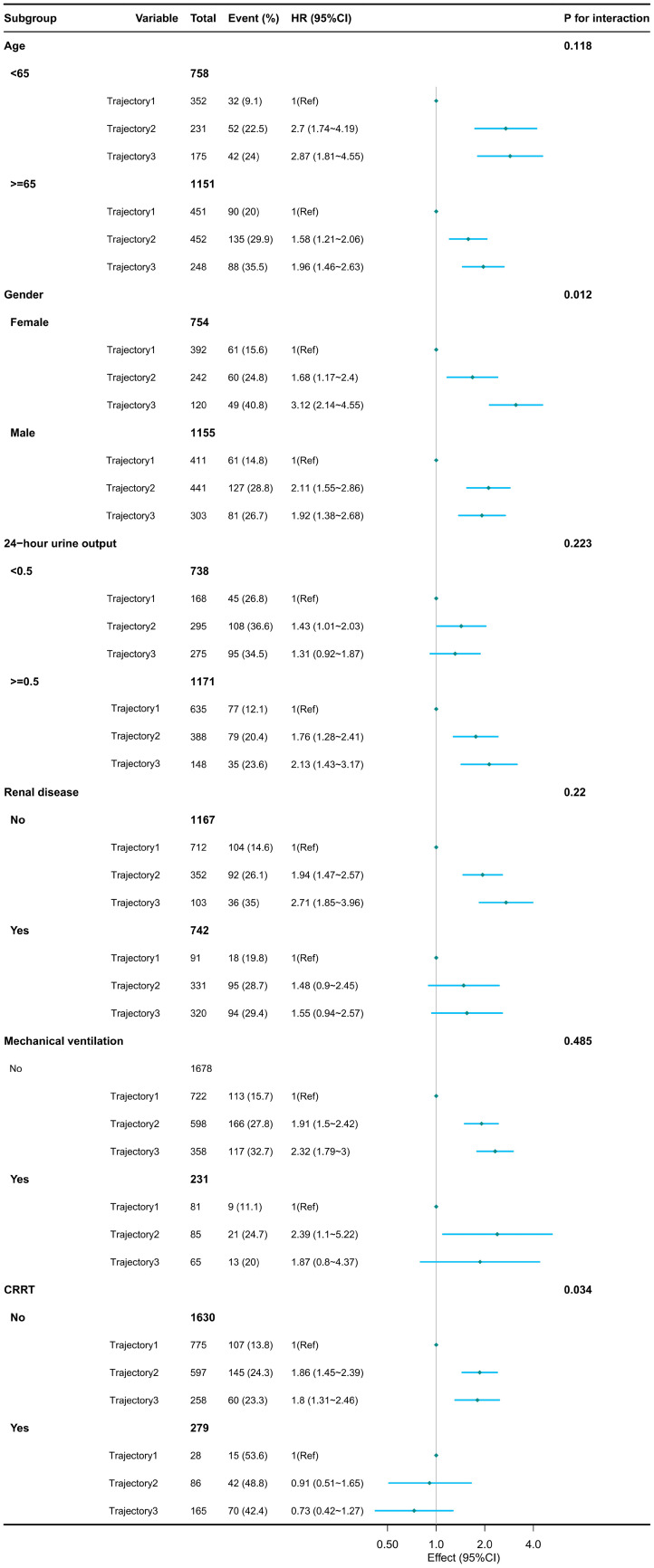
Forest plot of subgroup analyses evaluating the association between creatinine trajectory patterns and 28-day mortality.

In the subgroup analysis (**Figure 4**), the association between trajectory grouping and 28-day mortality remained consistent across most subgroups: relative to Trajectory 1, Trajectory 2 and Trajectory 3 generally exhibited a higher risk of mortality, with a more distinct increasing trend observed in subgroups including those without pre-existing kidney disease, those not receiving mechanical ventilation, and those with higher urine output. Interaction tests revealed no significant effect modification by age, urine output stratification, pre-existing kidney disease, or mechanical ventilation. In contrast, significant interactions were identified for gender (P = 0.012) and receipt of CRRT (P = 0.034): among patients not receiving CRRT, the mortality risk of Trajectory 2 and Trajectory 3 was significantly higher than that of Trajectory 1; whereas among patients receiving CRRT, the risk difference between Trajectory 2/3 and Trajectory 1 was not statistically significant. However, given the small sample size of Trajectory 1 in the CRRT subgroup and the potential indication of higher disease severity by CRRT, this interaction result warrants further investigation.

### 3.5 Mediation analysis

Mediation analysis (**Supplementary Table 4**) demonstrated a significant overall association between the average creatinine level on the first day of ICU admission and 28-day mortality (total effect: OR = 1.10, 95% CI 1.05–1.16, P < 0.001). Elevated creatinine was found to significantly increase the likelihood of CRRT utilization (a path: OR = 1.47, 95% CI 1.38–1.57, P < 0.001), and CRRT was significantly associated with 28-day mortality (b path: OR = 3.44, 95% CI 2.59–4.58, P < 0.001). Following the inclusion of CRRT in the model, the direct effect of creatinine on 28-day mortality was no longer statistically significant (c’ path: OR = 1.01, 95% CI 0.96–1.08, P = 0.638), indicating that CRRT may act as a partial mediator in the pathway linking creatinine trajectories to mortality; however, it may also serve as a marker of disease severity and reflect treatment decisions driven by clinical judgment.

### 3.6 Sensitivity analysis

In the sensitivity analysis leveraging serum creatinine trajectories collected during the first 7 days following ICU admission, analogous three-group trajectory patterns were identified. The model exhibited excellent classification performance, with AvePP ranging from 0.976 to 0.986 and OCC values spanning 74.387 to 189.429 (**Supplementary Table 5**). The identified trajectory patterns were generally consistent with those observed in the primary 72-hour analysis, thereby supporting the robustness of the trajectory classification framework (**Supplementary Figure 3**).

## 4 Discussion

In this study, we used GBTM for modeling longitudinal serum creatinine profiles among ICU patients who have both sepsis and T2DM, identifying three clinically meaningful trajectory patterns in the current sample of interest, namely; stable low-levels (42%) and medium high level fluctuating group (36%) groups, and a persistently high-level deteriorating group. The patients who were categorized into the persistently high level trajectory group had a significantly higher 28-day all-cause mortality(HR = 2.29, 95% CI: 1.78 - 2.94), but persisted even when fully adjusting for confounders (HR = 1.46, 95% CI: 1.04 - 2.05). A potential interaction between CRRT and creatinine trajectories was observed for mortality outcomes (interaction P = 0.034). However, given the potential for confounding by indication and small subgroup sample sizes, this finding should be interpreted as exploratory. CRRT may reflect both treatment selection and the severity of underlying disease, rather than a definitive effect modifier.

Peak creatinine is less sensitive than creatinine trajectories in capturing the temporal course and dynamic nature of renal dysfunction. In this study, different creatinine trajectories may indicate heterogeneous patterns of early renal dysfunction rather than directly proving specific biological mechanisms. A persistently high or worsening creatinine trajectory may be associated with more severe renal impairment, impaired renal reserve, and systemic inflammatory burden. In patients with sepsis and T2DM, several mechanisms may plausibly contribute to this association. Sepsis-induced systemic inflammation can aggravate endothelial dysfunction, while T2DM-related glomerular hyperfiltration, tubulointerstitial fibrosis, and reduced renal reserve may increase susceptibility to septic kidney injury (12–14). As previously reported, baseline creatinine levels in patients with T2DM may be related to renal functional reserve: low creatinine may be associated with sarcopenia, whereas increased creatinine often indicates a substantial reduction in glomerular filtration rate (15). Under septic conditions, these patients may therefore be more vulnerable to rapid deterioration in renal function (4). Furthermore, T2DM-associated metabolic dysregulation, including hyperglycemia and insulin resistance, may amplify sepsis-related oxidative stress and mitochondrial injury, thereby contributing to renal tubular epithelial cell dysfunction (16). Consistent with this possibility, patients in the persistently high creatinine trajectory group showed higher inflammatory marker levels, such as WBC, in our cohort. However, these findings should be interpreted as associations rather than direct evidence of microvascular injury or metabolic dysregulation (17–19).

The majority of previous AKI research has used either the highest creatinine value, or a change in creatinine over two days to define stages according to the KDIGO classification (20), but these static measures are not able to reflect the heterogeneity of renal function evolution (12). Re-defining AKI as a dynamic syndrome by using trajectory modeling, these results are consistent with several other studies: Takkavatakarn et al. divided sepsis-related AKI patients into eight creatinine profile clusters and found that the cluster labeled “severe AKI but with mild but sustained recovery” was at increased risk for acute kidney disease (AKD) (4). In addition, trajectory analysis for creatinine after cardiac surgery also validated “persistent moderate-to high level” as an independent predictor of late mortality (21). The novelty of this study lies in its focus on the high-risk sepsis-T2DM subpopulation, demonstrating that trajectory-based classification enables more granular risk stratification against the backdrop of pre-existing diabetic nephropathy. Notably, GBTM has also been applied to analyze dynamic changes in the UACR in T2DM patients, revealing associations between distinct trajectories and cardiovascular events—further validating the utility of trajectory analysis in managing diabetic complications (22).

In the stratified analysis for CRRT, patients with a high creatinine trajectory had a higher risk of death among those receiving CRRT than among those not receiving CRRT. This finding suggests that CRRT use may identify a subgroup of patients with more severe renal dysfunction and critical illness. Although CRRT may partly lie on the pathway between renal dysfunction and mortality, it is also strongly influenced by clinical decision-making and disease severity. Day 1 mean creatinine was associated with higher odds of initiating CRRT (OR = 1.47; 95% CI: 1.38–1.57), and CRRT was also associated with mortality (OR = 3.44; 95% CI: 2.59–4.58). The mediation analysis was therefore considered exploratory. Although the indirect pathway through CRRT was observed, this should not be interpreted as definitive causal evidence, given the observational design and the potential for confounding by indication. The decision to initiate CRRT is based on clinical conditions such as refractory metabolic derangements, electrolyte imbalance, fluid overload, and hemodynamic instability, all of which are themselves associated with poor prognosis (23). Thus, CRRT may simultaneously represent a therapeutic intervention, a marker of disease severity, and a potential intermediate factor. Differences in the timing, modality, and intensity of CRRT may also influence outcomes independently of creatinine trajectory. In sex-stratified analyses, higher creatinine trajectories were also associated with mortality among women. However, this subgroup finding should be interpreted cautiously because the analysis was exploratory and may have been affected by residual confounding and limited subgroup size. Potential biological explanations, such as sex-related differences in renal vascular function, endothelial vulnerability, or hormonal regulation, remain speculative and were not directly tested in the present study (24–26). Further studies are needed to validate whether sex modifies the relationship between creatinine trajectories and outcomes in patients with sepsis and T2DM.

Our study suggest that real-time tracking of creatinine curves during the first three days in the ICU can help to identify patient clusters at risk for mortality, who were diagnosed with sepsis and type two diabetes mellitus, thus creating an important therapeutic window for tailored renoprotection interventions - such as optimized fluid management and avoidance of nephrotoxic agents (9, 27). Further integration of trajectory-based classification into clinical decision support systems holds promise for enhancing precision monitoring of this patient population.

## 5 Limitations

This study has several limitations that warrant acknowledgment. First, as a retrospective single-center study leveraging the MIMIC-IV database, residual confounding cannot be fully eliminated despite rigorous multivariable adjustment. Second, information related to CRRT was extracted from electronic health records and coding systems, which may introduce information bias and indication-related confounding. Third, serum creatinine levels may be confounded by non-steady-state physiological conditions, including muscle wasting, fluid balance disturbances, and altered creatinine synthesis. Consequently, creatinine trajectories may not fully capture true renal function. Future studies incorporating complementary biomarkers such as cystatin C could enhance the accuracy of renal function assessment (28). Notably, group-based trajectory modeling is a data-driven approach that hinges on modeling assumptions, including the number of trajectory groups and polynomial specifications. Alternative model selections may yield divergent classification results, and a certain degree of misclassification remains unavoidable. Thus, the identified trajectory groups should be interpreted as statistical constructs rather than definitive biological phenotypes. Furthermore, Sepsis was identified via ICD-9/10-CM diagnostic codes rather than prospective Sepsis-3 clinical adjudication, which may have introduced classification bias. In addition, the requirement for repeated creatinine measurements prior to ICU admission may have preferentially enrolled patients with more intensive monitoring or greater disease severity, potentially introducing selection bias. Finally, given that this study was conducted using a single intensive care unit database, the external generalizability of the findings to other populations and healthcare systems may be limited. External validation in multicenter prospective cohorts is therefore imperative. Multi-center prospective studies should be conducted in future for validation of the trajectory model, and further exploration of how to integrate multi-omics markers (e.g., metabolites, cytokines) to improve risk prediction (29, 30).

## 6 Conclusion

In critically ill patients with sepsis complicated by T2DM, early changes in serum creatinine are significantly associated with 28-day mortality. Patients with persistently elevated creatinine levels exhibit a higher risk of death compared to those with stable creatinine levels. The requirement for continuous renal replacement therapy may partially account for the association between early renal dysfunction and mortality.

## Data Availability

The original contributions presented in the study are included in the article/**Supplementary Material**. Further inquiries can be directed to the corresponding authors.
