# Supplementary material for: Trajectory models of serum creatinine and 28-day mortality in critically ill patients with sepsis complicated by type 2 diabetes mellitus: a cohort study
Source: Front Endocrinol (Lausanne). 2026 Jun 23;17:1822280. doi: 10.3389/fendo.2026.1822280 (PMC13337474; doi:10.3389/fendo.2026.1822280)
Supplement: Supplementary file 1 [file Table1.docx]

Supplementary Material

# Supplementary Figures and Tables

## Supplementary Figures

**
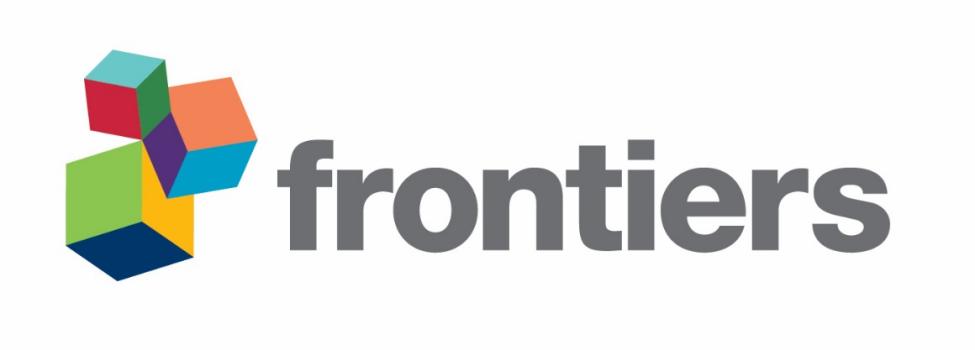
**

**Supplementary Figure 1.** Variable importance identified by the Boruta feature selection algorithm.

**Supplementary Figure 2.** Restricted cubic spline analysis of the association between creatinine and 28-day mortality.

**Supplementary Figure 3.** Trajectory trends of serum creatinine within 7 days of ICU admission.

## Supplementary Tables

**Supplementary Table 1.** ICD Codes for sepsis and T2DM.

| **Disease name** | **ICD** |
| --- | --- |
| Sepsis | **ICD-9 Codes**  670.20, 670.22,670.24,771.81,995.91,995.92  **ICD-10 Codes**  A02.1,A22.7,A26.7,A32.7,A40,A40.0,A40.1,A40.3,A40.8,A40.9,A41,A41.0,A41.01,A41.02,A41.1,A41.2,A41.3,A41.4,A41.5,  A41.50,A41.51,A41.52,A41.53,A41.59,A41.8,A41.81,A41.89,A41.9,A42.7,A54.86,B37.7,O03.37,O03.87,O04.87,O07.37,O08.82,O85,O86.04,P36,P36.0,P36.1,P36.10,P36.19,P36.2,P36.3,P36.30,P36.39,P36.4,P36.5,P36.8,P36.9,R65.2,R65.20,R65.21,T81.44,T81.44XA,T81.44XD,T81.44XS |
| T2DM | **ICD-9 Codes** (250.xx)  250.00,250.01,250.02,250.03,250.10,250.11,250.12,250.13,250.20,250.21,250.22,250.23,  250.30,250.31,250.32,250.33,250.40,250.41,250.42,250.43,250.50,250.51,250.52,250.53,  250.60,250.61,250.62,250.63,250.70,250.71,250.72,250.73,250.80,250.81,250.82,250.83,  250.90,250.91,250.92,250.93  **ICD-10 Codes**  E11.x |

**Supplementary Table 2.** Evaluation of Classification Quality and Model Fit for Group-Based Trajectory Models.

| **value** | **group1** | **group2** | **group3** |
| --- | --- | --- | --- |
| Prob | 0.414 | 0.363 | 0.223 |
| Group rate | 0.421 | 0.358 | 0.222 |
| AvePP | 0.957 | 0.945 | 0.969 |
| OCC | 31.36 | 30.162 | 109.768 |

**Supplementary Table 3.** Comparison of Candidate Group-Based Trajectory Models and Classification Diagnostics.

| **Model** | **BIC** | **Group rate** | **AvePP range** | **OCC range** |
| --- | --- | --- | --- | --- |
| 2-group | 15576.12 | 65.5%, 34.5% | 0.969–0.981 | 27.099–59.361 |
| 3-group | 13088.87 | 42.1%, 35.8%, 22.2% | 0.945–0.969 | 30.162–109.768 |
| 4-group | 11760.22 | 32.6%, 30.0%, 26.8%, 10.6% | 0.926–0.958 | 26.268–187.236 |

**Supplementary Table 4.** Mediation analysis regression models.

| **Model / Path** | **Predictor** | **OR (95% CI)** | **P -value** |
| --- | --- | --- | --- |
| (1) 28-day mortality (Total effect, c) | creat1 | 1.10 (1.05–1.16) | <0.001 |
| (2) CRRT use (a path) | creat1 | 1.47 (1.38–1.57) | <0.001 |
| (3) 28-day mortality (Direct effect, c′) | creat1 | 1.01 (0.96–1.08) | 0.638 |
| (3) 28-day mortality (Mediator effect, b) | CRRT | 3.44 (2.59–4.58) | <0.001 |

Model (1) estimates the total effect of day-1 serum creatinine (creat1) on 28-day mortality (c).

Model (2) estimates the effect of creat1 on CRRT use (a).

Model (3) includes both creat1 and CRRT to estimate the direct effect of creat1 (c′) and the mediator effect of CRRT (b).

**Supplementary Table 5.** Evaluation Metrics for Group-Based Trajectory Modeling of Serum Creatinine Within 7 Days of ICU Admission.

| **value** | **group1** | **group2** | **group3** |
| --- | --- | --- | --- |
| prob | 0.379 | 0.355 | 0.266 |
| CI lower | 0.37 | 0.348 | 0.259 |
| CI upper | 0.388 | 0.363 | 0.272 |
| group_rate | 0.38 | 0.354 | 0.266 |
| AvePP | 0.985 | 0.976 | 0.986 |
| OCC | 106.98 | 74.387 | 189.429 |
