# Supplementary figures and images for: Trajectory models of serum creatinine and 28-day mortality in critically ill patients with sepsis complicated by type 2 diabetes mellitus: a cohort study
Source: Front Endocrinol (Lausanne). 2026 Jun 23;17:1822280. doi: 10.3389/fendo.2026.1822280 (PMC13337474; doi:10.3389/fendo.2026.1822280)

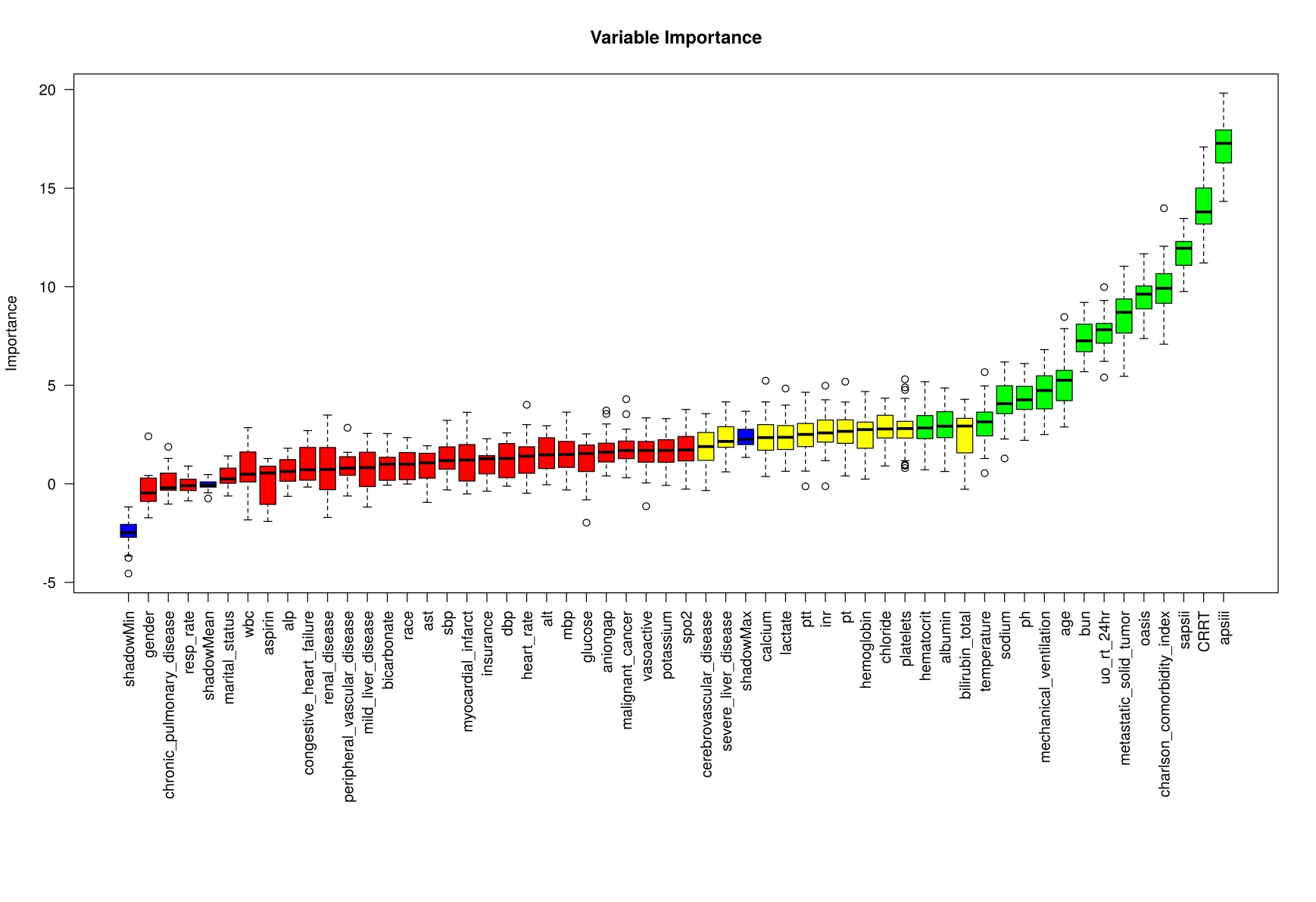

Supplement: Supplementary file 2 [file Image1.jpeg]

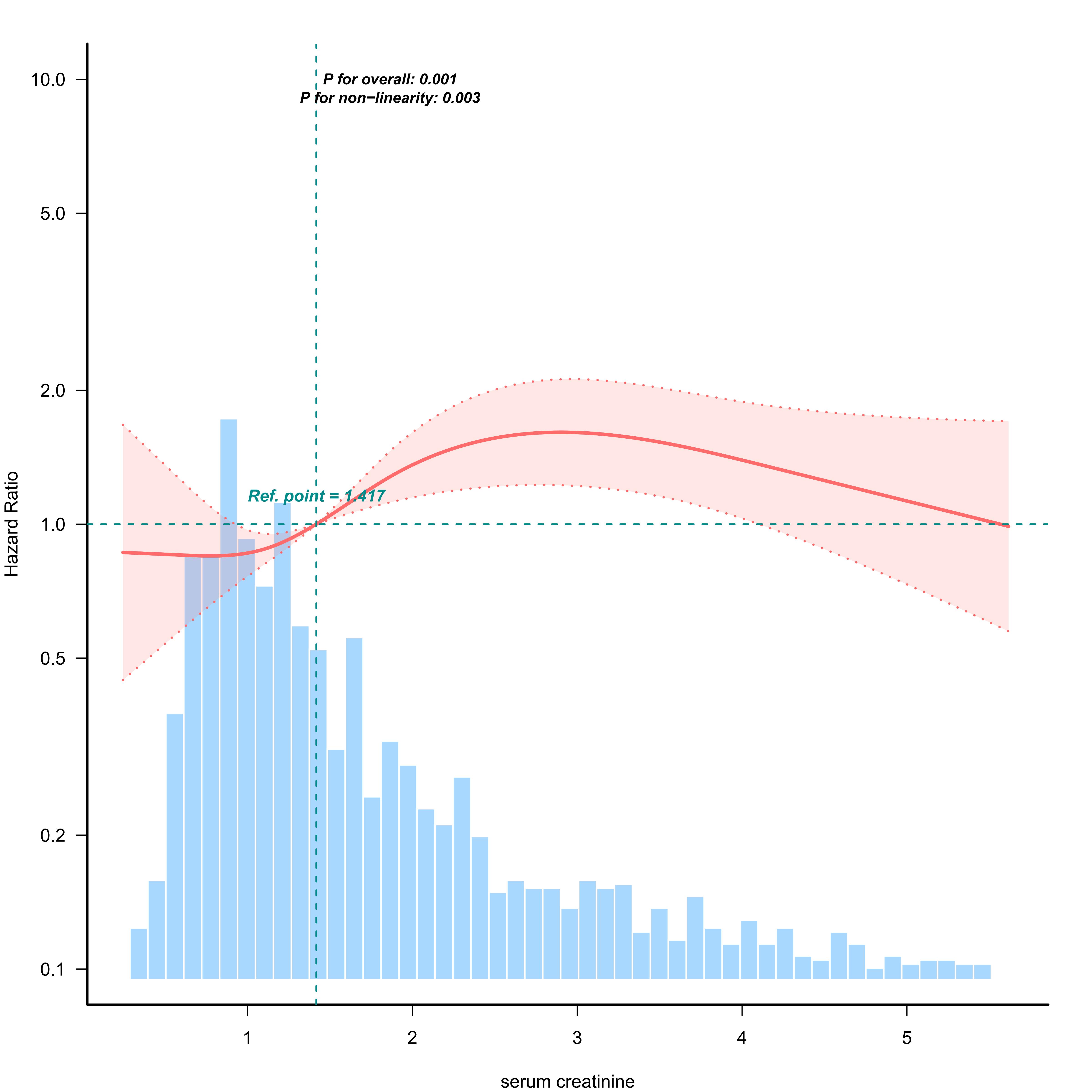

Supplement: Supplementary file 3 [file Image2.jpeg]

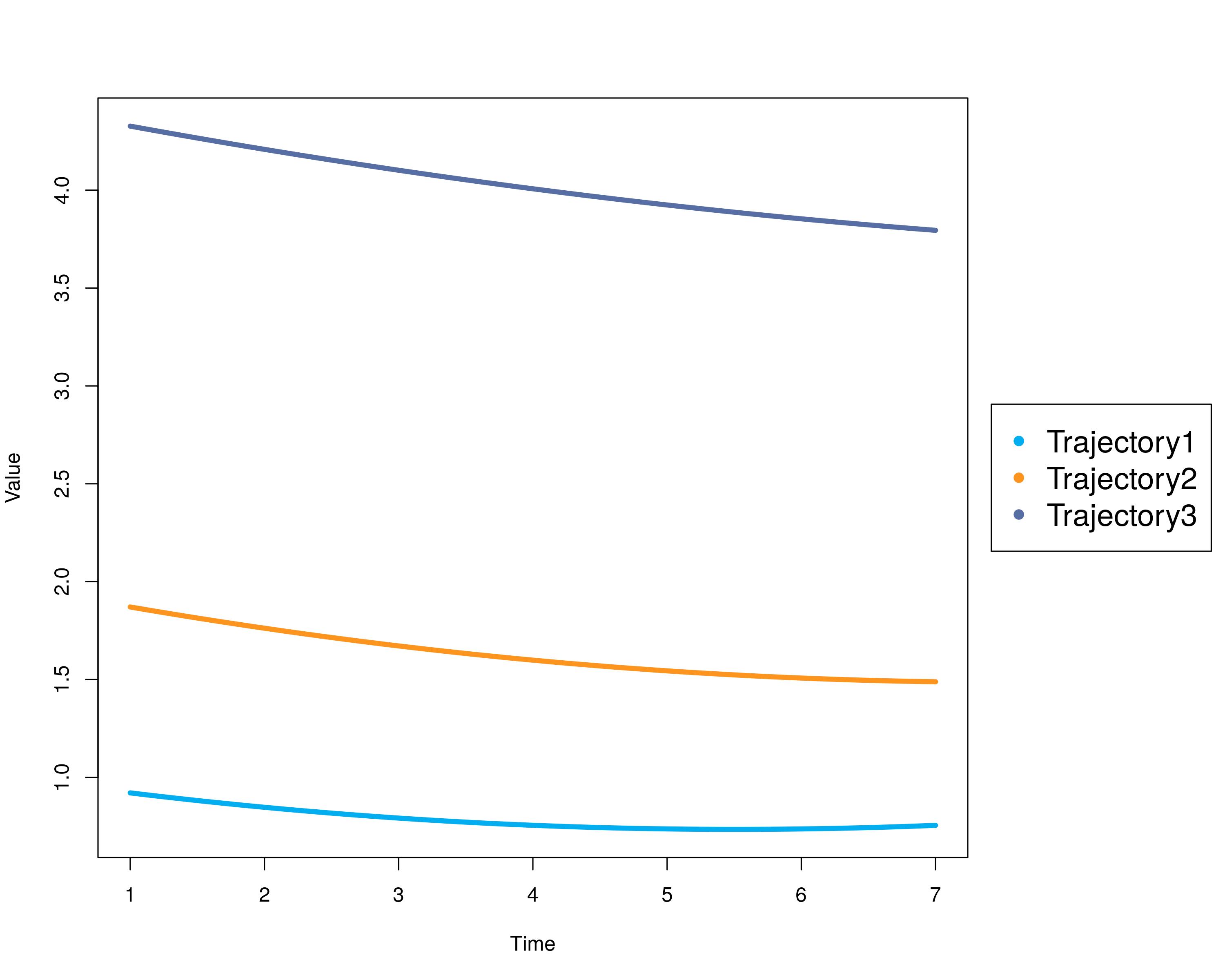

Supplement: Supplementary file 4 [file Image3.jpeg]
